# A 10-Year Behavioral and Social Sciences Research Agenda for ADRD: Reflection on a New National Academies Report

**DOI:** 10.1093/geroni/igab046.005

**Published:** 2021-12-17

**Authors:** Tia Powell, David Reuben, Vincent Mor, Deborah Blacker, Molly Checksfield

**Affiliations:** 1 Montefiore Medical Center, Bronx, New York, United States; 2 UCLA, Los Angeles, California, United States; 3 Providence, Rhode Island, United States; 4 Mass General Hospital, Charlestown, Massachusetts, United States; 5 National Academies of Sciences, Engineering and Medicine, Washington, District of Columbia, United States

## Abstract

The National Academies of Sciences, Engineering, and Medicine (NASEM) was charged with developing a ten-year agenda for research in the behavioral and social sciences that would substantially contribute to reducing the impact of Alzheimer’s disease and related dementias (AD/ADRD). The report, expected to be publicly released in June 2021, has been developed by a committee of individuals with expertise across a range of disciplines and fields, including dementia research. The committee was charged with assessing the role of the social and behavioral sciences in reducing the impact of dementia. The committee held several evidence-gathering sessions, reviewed published literature, commissioned several papers, and engaged individuals living with dementia and caregivers as a part of an Advisory Panel to the committee. This presentation will engage attendees in a discussion about the ten-year behavioral and social science research agenda related to dementia produced by this NASEM committee.

